# Protective effects of melatonin against physical injuries to testicular tissue: A systematic review and meta-analysis of animal models

**DOI:** 10.3389/fendo.2023.1123999

**Published:** 2023-01-31

**Authors:** Niloofar Dehdari Ebrahimi, Sara Shojaei-Zarghani, Ehsan Taherifard, Sanaz Dastghaib, Shima Parsa, Nasim Mohammadi, Fatemeh Sabet Sarvestani, Zahra Moayedfard, Nima Hosseini, Heidar Safarpour, Alireza Sadeghi, Negar Azarpira, Ali Reza Safarpour

**Affiliations:** ^1^ Transplant Research Center, Shiraz University of Medical Sciences, Shiraz, Iran; ^2^ Gastroenterohepatology Research Center, Shiraz University of Medical Sciences, Shiraz, Iran; ^3^ Endocrinology and Metabolism Research Center, Shiraz University of Medical Sciences, Shiraz, Iran; ^4^ Department of Tissue Engineering and Cell Therapy, School of Advanced Technologies in Medicine, Shiraz University of Medical Sciences, Shiraz, Iran; ^5^ Health Policy Research Center, Institute of Health, Shiraz University of Medical Sciences, Shiraz, Iran

**Keywords:** infertility, melatonin, rodents, oxidative stress, ischemia, reperfusion, heat

## Abstract

**Background:**

Modern societies face infertility as a global challenge. There are certain environmental conditions and disorders that damage testicular tissue and may cause male infertility. Melatonin, as a potential antioxidant, may protect testicular tissue. Therefore, we conducted this systematic review and meta-analysis to evaluate the effects of melatonin in animal models against physical, heat, and ischemic damage to the testicular tissue.

**Methods:**

PubMed, Scopus, and Web of Science were systematically searched to identify animal trials evaluating the protective effect of melatonin therapy on rodent testicular tissue when it is exposed to physical, thermal, ischemic, or hypobaric oxygen stress. Random-effect modeling was used to estimate the standardized mean difference and 95% confidence intervals based on the pooled data. Additionally, the Systematic Review Centre for Laboratory Animal Experimentation (SYRCLE) tool was used to assess the risk of bias. The study protocol was prospectively registered in PROSPERO (CRD42022354599).

**Results:**

A total of 41 studies were eligible for review out of 10039 records. Studies employed direct heat, cryptorchidism, varicocele, torsion-detorsion, testicular vascular occlusion, hypobaric hypoxia, ischemia-reperfusion, stress by excessive or restraint activity, spinal cord injury, and trauma to induce stress in the subjects. The histopathological characteristics of testicular tissue were generally improved in rodents by melatonin therapy. Based on the pooled data, sperm count, morphology, forward motility, viability, Johnsen’s biopsy score, testicular tissue glutathione peroxidase, and superoxide dismutase levels were higher in the melatonin treatment rodent arms. In contrast, the malondialdehyde level in testicular tissue was lower in the treatment rodent arms. The included studies suffered from a high risk of bias in most of the SYRCLE domains.

**Conclusion:**

This study concludes that melatonin therapy was associated with improved testicular histopathological characteristics, reproductive hormonal panel, and tissue markers of oxidative stress in male rodents with physical, ischemic, and thermal testicular injuries. In this regard, melatonin deserves scientific investigations as a potential protective drug against rodent male infertility.

**Systematic review registration:**

https://www.crd.york.ac.uk/PROSPERO/, identifier CRD42022354599.

## 1 Introduction

The pathophysiology of male infertility is caused by a number of variables, including genetics and epigenetic changes, hormonal imbalances, environmental influences, and physical injuries like varicocele, cryptorchidism, and testicular torsion (1, 2). Some of these factors change the balance between the generation of free-radical species and the antioxidant defense system, which in turn disrupts functional male fecundity. Reactive oxygen species (ROS) are generally crucial for some common physiological processes like spermatogenesis, sperm capacitation, and the acrosome reaction; however, an increase in ROS production leads to “oxidative stress” (3, 4), which harms cells by inducing oxidative damages like lipid peroxidation, DNA damage, and protein misfolding resulting to abnormal semen parameters (5). Utilizing antioxidant supplements, such as melatonin, zinc, coenzyme Q10 (CoQ10), omega-3 fatty acids, vitamin E, and L-carnitine, as novel treatment strategies to address male infertility diseases has recently drawn increasing attention (6–8).

Melatonin, the sleep-wakefulness hormone, was initially discovered by Aaron Lerner in the pineal gland of a bovine in 1958 (9). Once believed that it only is secreted by the pineal gland, melatonin is now understood to be produced throughout the body in various tissues, including the cardiovascular, endocrine, immunological, male reproductive, skin, and gastrointestinal tract systems (10, 11). As an endogenous indole amine, melatonin plays a crucial role in many biological processes, like circadian rhythm, redox homeostasis, epigenetic regulation, body temperature regulation, fetal development, local and general immunity, and reproductive physiology (5, 12, 13). Melatonin has been extensively explored for its antioxidant properties (14–17). Melatonin can pass through the blood-testis barrier and is capable of entering testis cells (18). Melatonin has direct and indirect antioxidant properties. As an electron-rich molecule, it can negate free radicals, making stable products that are able to be excreted in urine. Melatonin’s antioxidant mechanism involves free-radical scavenger cascade indicating efficiency of secondary and tertiary metabolites. Furthermore, melatonin indirectly acts by stimulating antioxidant enzymes (19–21). In fact, it can modulate the mRNA levels and activity of some well-known antioxidants such as Glutathione Peroxidase (GPx), ascorbate, and superoxide dismutase (SOD) (15, 22). It plays a role in the production and secretion of testosterone by Leydig cells and increases the responsiveness of Sertoli cells to Follicle-Stimulating Hormone (FSH) during testis development and growth (23, 24). The detrimental disorders of unilateral testicular damages (such as undescended testis and torsion), which influence morphometric, spermatogenesis, and oxidative parameters, could be reversed by melatonin as an antioxidant (25, 26). Up to now, it’s widely recognized that melatonin has different effective roles in the male reproductive system, which exerts these effects directly (non-receptor mediated) or indirectly (receptor-mediated pathways) (27, 28). In humans, melatonin has two types of high-affinity G-protein-coupled receptors called melatonin type 1 and 2 receptors (MT1 and MT2) (29). In the male reproductive system, melatonin, through the effect of MT1, MT2, and retinoic acid receptor-related orphan receptor/retinoid Z receptor (ROR/RZR) implicates in proliferation (such as spermatogonial stem cells-SSCs), differentiation (such as spermatogonia to spermatids) and metabolic functions (23, 30, 31). On the other hand, melatonin acts directly as ROS and reactive nitrogen scavenger (RNS) according to its structure that had been suggested to be an electron donor (32, 33). Melatonin is believed to have an anti-apoptotic effect in the testes by decreasing mitochondrial-related apoptosis and ROS-related mitochondrial damage (34, 35).

Animal models have gained interest due to the limitations of human studies in reproductive research, such as ethical limitations in administering drugs and performing biopsies, and the long and ambiguous period of disease development and progression. As a result, we decided to systematically review the literature for rodent animal studies that used, evaluated, and reported melatonin and its protective effects on male genital systems against physical injuries.

## 2 Materials and method

This systematic review and meta-analysis was conducted following The Preferred Reporting Items for Systematic reviews and Meta-Analyses (PRISMA) statement (36), and the protocol is registered in the International Prospective Register of Systematic Reviews (PROSPERO: CRD42022354599).

### 2.1 Key question

This review aimed to investigate the protective effects of exogenous melatonin on the parameters of male reproductive function against physical, heat, or ischemic injuries compared to placebo in rodent subjects. We asked if melatonin therapy (whether before or after the induction of stress) prevents oxidative pathways in testicular cells.

### 2.2 Data sources and searches

Two reviewers (AS and SP) conducted a comprehensive search in PubMed, Scopus, and Web of Science for records from January 1, 1970, until September 9, 2022, for “melatonin”, “testicular function”, and their equivalents as keywords. The search strategies comprised a combination of Medical Subject Headings (MeSH) or their equivalent (where available), keywords, truncations, and boolean operators. A manual backward and forward citation search was also done for all the included studies. The detailed search strategy is provided in **Supplementary Material 1**.

### 2.3 Study selection and eligibility criteria

Firstly, duplicate records were removed electronically. Then, using the Rayyan online tool for managing systematic reviews (37), four reviewers (NDE, SP, NM, and AS) independently screened titles and abstracts, followed by the full-text screening of the identified records for eligibility criteria. Disagreements were resolved with discussion. Studies were included if they satisfied the following criteria: (1) controlled animal studies, (2) the population was rodents that were exposed to physical, electrical, ischemic, or thermal injuries as oxidative stress to the testicular tissue, (3) at least one intervention group received melatonin regimen, (4) at least one control group with similar stress, and (5) reported major hallmarks of testicular tissue (histopathologic, biochemical, and sperm analyses).

The exclusion criteria were: (1) *in-vitro* and ex-vivo studies, (2) non-rodent animals with other types of stresses (radiation, chemotherapy, toxins, and metabolic), (3) combination therapy of melatonin with other drugs, (4) treatment using derivatives of melatonin, (5) healthy controls without stress, and (6) reported irrelevant outcomes. Also, reviews, letters, and human trails were excluded from the review. Our search was not restricted by language.

### 2.4 Data extraction and risk of bias assessment

Two reviewers (ET and FSS) independently extracted the favorable data into Excel spreadsheets. Any disagreements were resolved by discussion and involvement of a third author (AS). Structured forms were used for data extraction of the following contents: (1) study characteristics (first author, publication year, and country), (2) population characteristics (species, age, sample size, and type of stress), (3) melatonin dose, duration, and route, (4) time of assessment of outcome, (5) tissue and plasma biochemical indices, and (6) histopathological characteristics. For missing data, we contacted the first or corresponding author and waited for a response for at least one month.

We assessed the risk of bias in the studies using the Systematic Review Centre for Laboratory animal Experimentation (SYRCLE) tool for animal intervention studies (38). Two authors (NDE and ET) independently reviewed each article and classified it as high, low, or unclear for each bias domain. Disagreements were resolved *via* consensus or by a third (AS) reviewer if necessary.

### 2.5 Data synthesis and statistical analysis

Data were analyzed using Stata MP Version 16 (StataCorp, College Station, TX, USA), and a p-value < 0.05 was considered statistically significant. Under a random-effects model (DerSimonian-Laird method), the pooled effect sizes were reported as standardized mean difference (Glass’s Δ) and 95% confidence interval (39). The statistical heterogeneity was examined using Cochran’s Q statistic, p-value, and I-squared. I-squared was employed to qualify heterogeneity as “perhaps not important”, “moderate heterogeneity”, “substantial heterogeneity”, and “considerable heterogeneity” if I-squared values were 0-40%, 30-60%, 50-90%, and 75-100%, respectively (40). Subgroup analyses were implemented only if three or more studies were available for each subgroup to identify possible sources of heterogeneity. In case of missing data, if crucial, studies were removed from the analysis. Also, funnel plots were used to detect visual asymmetry in publications when at least ten studies were available for the outcome (40).

## 3 Results

### 3.1 Search results

**Figure 1:**
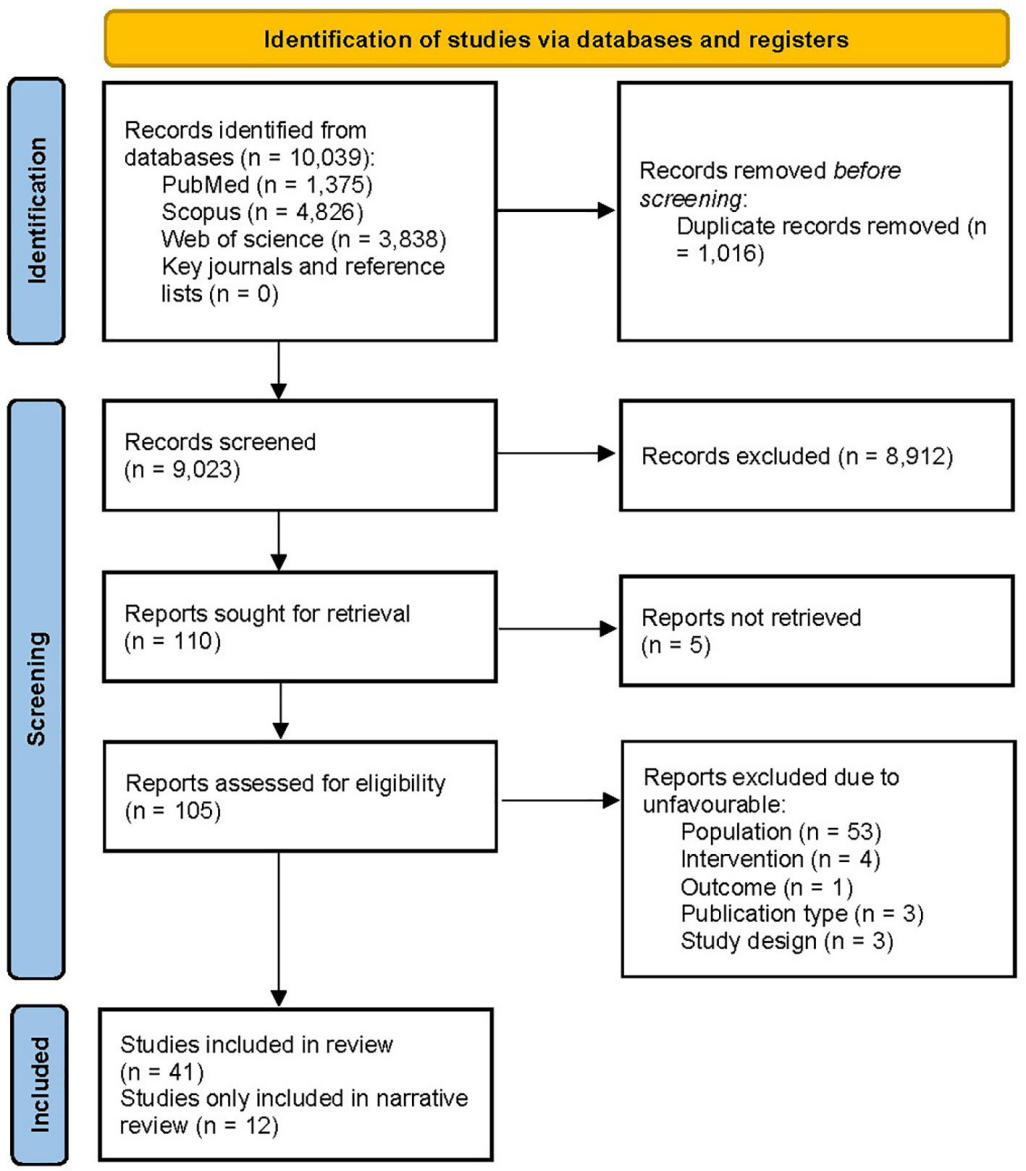
PRISMA flow diagram illustrating the process of selection of the studies.

The PRISMA flow diagram of the literature search is presented in **Figure 1**. The systematic search yielded 10,039 records, while manual citation searching yielded no additional studies. The database searching included PubMed (n=1,375), Web of Science (n=3,838), and Scopus (n=4,826). 1,016 records were removed using automatic duplicate detection. Title and abstract screening was conducted on 9,023 records, and 110 studies were sought for retrieval. With the exclusion of 5 studies that we failed to retrieve (41–45), 105 articles were assessed for eligibility. A total of 64 articles were excluded due to ineligible population (n=53), design (n=3), intervention (n=4), outcome (n=1), and publication type (n=3). Finally, 41 articles were eligible for the study; 12 (46–57) were only included in narrative data synthesis. Two studies were on the same subject population; thus, for data synthesis, they were treated as one (47, 51). Despite our effort to contact the authors, 8 studies contained missing values; therefore, they were excluded from the analysis (47, 52–55, 57–59).

### 3.2 Study characteristics

**Figure 2:**
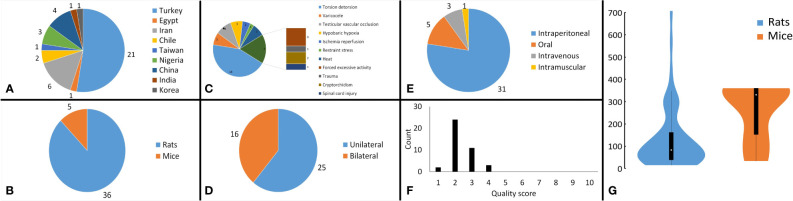
Analysis of study characteristics. Pie chart of **(A)** the countries that studies were published from, **(B)** the subject rodents across the studies, **(C)** the stress mechanism used in the studies, **(D)** the side that stresses were induced in the subjects, **(E)** the route of melatonin administration across the studies, **(F)** bar chart, illustrating the distribution of the methodological quality scores across the studies, and **(G)** violin plot demonstrating the distribution of overall cumulative dosages that were administered to the rodents (mg/kg).

Included studies were published between 2000 and 2022 in English. Rats (n=36) (25, 46–54, 56, 58–82) and mice (n=5) (55, 57, 83–85) were the subjects in the included studies. Studies employed direct heat (n=3) (82, 84, 85), cryptorchidism (n=2) (58, 72), varicocele (n=3) (60, 73, 77), torsion-detorsion (n=18) (25, 46, 48, 49, 54, 56, 59, 62–66, 74, 75, 78–81), testicular vascular occlusion (n=4) (52, 53, 61, 67), hypobaric hypoxia (n=3) (47, 51, 55), ischemia-reperfusion (n=2) (50, 76), stress by excessive (n=3) (68, 69, 71) or restraint (n=1) (83) activity, spinal cord injury (n=1) (57), and trauma (n=1) (70). Studies induced the stress mechanisms bilaterally (n=16) (47, 48, 50, 51, 55, 57, 58, 68, 69, 71, 72, 75, 76, 82–85) and unilaterally (n=25) (25, 46, 49, 52–54, 56, 58–67, 70, 73, 74, 77–81). Mice aged between 6 - 12 weeks and rats aged between 6 – 24 weeks. Studies administered melatonin intraperitoneal (n=31) (25, 46, 48, 49, 52–54, 56–60, 62, 63, 65–71, 73, 75, 77–81, 83–85), oral (n=5) (47, 51, 55, 61, 72, 82), intravenous (n=3) (50, 64, 76), and intramuscular (n=1) (74). Characteristics of the included articles are summarized in **Figure 2**. The dose of melatonin therapy, descriptive histopathological findings, duration of stress induction, and outcomes assessment ranged significantly between the studies presented in **Table 1** and **Supplementary Materials 2**.

**Table 1:** Basic characteristics of the included studies.

First author [year]	Country	Rodent species	Injury (location)	n/intervention, control	Age of subjects	Melatonin per dose	Route of intervention	SYRCLE score
**Abasiyanik [2004]** (80)	Turkey	Rats	Torsion detorsion (Unilateral)	17, 12	N/M	N/M	IP	2
**Abo El Gheit [2021]** (60)	Egypt	Rats	Varicocele (Unilateral)	13, 13	12 weeks	10 mg/kg	IP	3
**Aktas [2011]** (46)	Turkey	Rats	Torsion detorsion (Unilateral)	21, 7	N/M	N/M	IP	1
**Asghari [2016]** (61)	Iran	Rats	Testicular vasculature occlusion (Unilateral)	10, 10	N/M	3 mg/kg	Oral	3
**Bustos-Obregón [2010] & Hartley [2009]** (47, 51)	Chile	Rats	Intermittent hypobaric hypoxia (Bilateral)	48, 48	8-12 weeks	10 mg/kg	Oral	2
**Chen [2021]** (48)	Taiwan	Rats	Torsion detorsion (Bilateral)	6, 6	N/M	50 mg/kg	IP	2
**Duru [2007]** (62)	Nigeria	Rats	Torsion detorsion (Unilateral)	60, 30	N/M	N/M	IP	2
**Ekici [2012]** (63)	Turkey	Rats	Torsion detorsion (Unilateral)	6, 6	12 weeks	10 mg/kg	IP	3
**Erdemir [2008]** (49)	Turkey	Rats	Torsion detorsion (Unilateral)	10, 10	22- 24 weeks	N/M	IP	3
**Esrefoglu [2004]** (50)	Turkey	Rats	Ischemia reperfusion (Bilateral)	8, 8	N/M	10 mg/kg	IV	2
**Gul [2018]** (64)	Turkey	Rats	Torsion detorsion (Unilateral)	12, 12	90 days	40 mg/kg	IV	3
**Guo [2017]** (83)	China	Mice	Restraint stress (Bilateral)	10, 10	6 weeks	10 mg/kg	IP	2
**Gürbilek [2000]** (81)	Turkey	Rats	Torsion detorsion (Unilateral)	12, 12	N/M	50 mg/kg	IP	2
**Haldera [2020]** (82)	India	Rats	Heat (Bilateral)	6, 6	11-12 weeks	10 mg/kg	Oral	3
**Jeong [2010]** (65)	Korea	Rats	Torsion detorsion (Unilateral)	16, 12	6 and 10 weeks	N/M	IP	2
**Kanter [2010]** (66)	Turkey	Rats	Torsion detorsion (Unilateral)	8, 8	16 weeks	N/M	IP	3
**Koksal [2012]** (67)	Turkey	Rats	Testicular vasculature occlusion (Unilateral)	16, 16	N/M	N/M	IP	2
**Kurcer [2008]** (53)	Turkey	Rats	Testicular vasculature occlusion (Unilateral)	N/M	8 weeks	N/M	IP	2
**Kurcer [2010]** (52)	Turkey	Rats	Testicular vasculature occlusion (Unilateral)	N/M	8 weeks	N/M	IP	3
**Mahmudi [2022]** (68)	Iran	Rats	Forced treadmill exercise (Bilateral)	8, 8	N/M	10 mg/kg/week	IP	1
**Minaii [2013]** (69)	Iran	Rats	Forced swimming test (Bilateral)	12, 12	N/M	10 mg/kg/week	IP	3
**Mirhoseini [2017]** (25)	Iran	Rats	Torsion detorsion (Unilateral)	8, 8	N/M	N/M	IP	2
**Mirhoseini [2019]** (70)	Iran	Rats	Trauma (Unilateral)	16, 8	N/M	N/M	IP	2
**Moayeri [2017]** (71)	Iran	Rats	Forced swimming test (Bilateral)	10, 10	N/M	10 mg/kg/week	IP	2
**Olayaki [2017]** (72)	Nigeria	Rats	Cryptorchidism (Bilateral)	12, 6	N/M	4 and 10 mg/kg	Oral	2
**Onur [2004]** (73)	Turkey	Rats	Varicocele (Unilateral)	20, 10	12-14 weeks	5 and 10 mg/kg	IP	2
**Ozturk [2003]** (74)	Turkey	Rats	Torsion detorsion (Unilateral)	10, 10	N/M	N/M	IM	2
**Parlaktas [2014]** (75)	Turkey	Rats	Torsion detorsion (Bilateral)	7, 7	20-24 weeks	N/M	IP	3
**Qin [2021]** (84)	China	Mice	Heat (Bilateral)	5, 5	9 weeks	20 mg/kg	IP	2
**Saalu [2006]** (58)	Nigeria	Rats	Cryptorchidism (Bilateral and unilateral)	16, 16	N/M	0.7 mg/kg	IP	2
**Sahna [2006]** (76)	Turkey	Rats	Ischemia reperfusion (Bilateral)	6, 6	8 weeks	10 mg/kg	IV	4
**Sekmenli [2016]** (54)	Turkey	Rats	Torsion detorsion (Unilateral)	7, 7	12 weeks	17 mg/kg	IP	4
**Semercioz [2003]** (77)	Turkey	Rats	Varicocele (Unilateral)	10, 10	12-14 weeks	10 mg/kg	IP	3
**Semercioz [2017]** (59)	Turkey	Rats	Torsion detorsion (Unilateral)	10, 10	N/M	3 mg/kg	IP	2
**Vargas [2011]** (55)	Chile	Mice	Intermittent and continuous hypobaric hypoxia (Bilateral)	16, 16	12 weeks	10 mg/kg	Oral	2
**Yildirim [2006]** (56)	Turkey	Rats	Torsion detorsion (Unilateral)	14, 7	N/M	N/M	IP	2
**Yuan [2016]** (57)	China	Mice	Spinal cord injury (Bilateral)	N/M	8 weeks	N/M	IP	2
**Yurtçu [2008]** (78)	Turkey	Rats	Torsion detorsion (Unilateral)	10, 10	7.5 weeks	17 mg/kg	IP	4
**Yurtçu [2009]** (79)	Turkey	Rats	Torsion detorsion (Unilateral)	20, 10	N/M	17 mg/kg	IP	2
**Zhang [2020]** (85)	China	Mice	Heat (Bilateral)	60, 30	10-12 weeks	20 mg/kg	IP	2

N/M, not mentioned; IP, Intraperitoneal; IV, Intravenous; IM, Intramuscular; SYRCLE, Systematic Review Centre for Laboratory Animal Experimentation.

### 3.3 Outcomes

**Figure 3:**
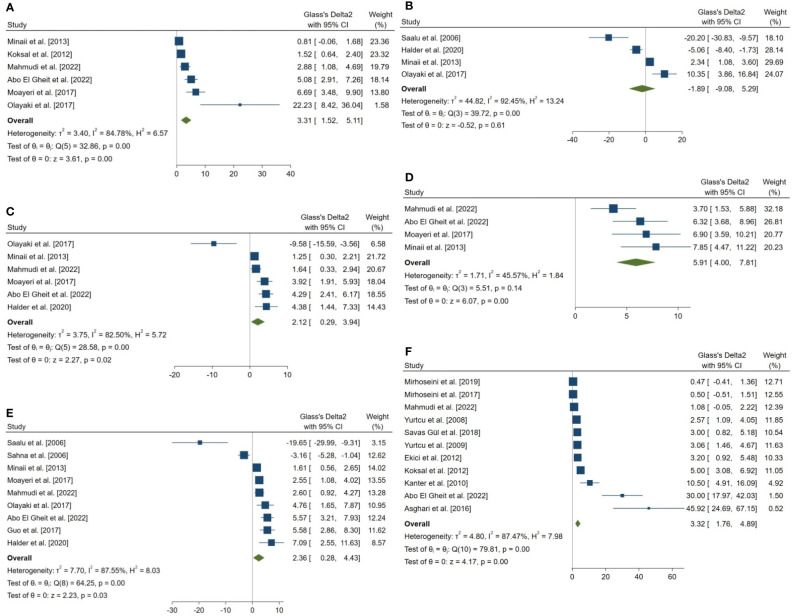
Forest plots for the overall pooled effects sizes of sperm parameters. **(A)** normal morphology (n=6), **(B)** total motility (n=4), **(C)** viability (n=6), **(D)** forward progressive motility (n=4), **(E)** total count (n=9), **(F)** Johnsen’s biopsy score (n=11).

**Figure 4:**
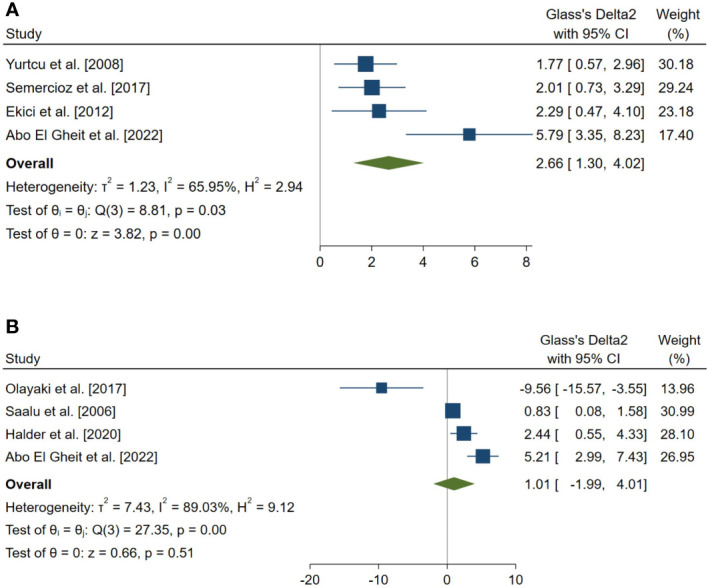
Forest plots for the overall pooled effects sizes of serum level of reproductive hormones. **(A)** Serum Inhibin-B (n=4), **(B)** testosterone (n=4).

**Figure 5:**
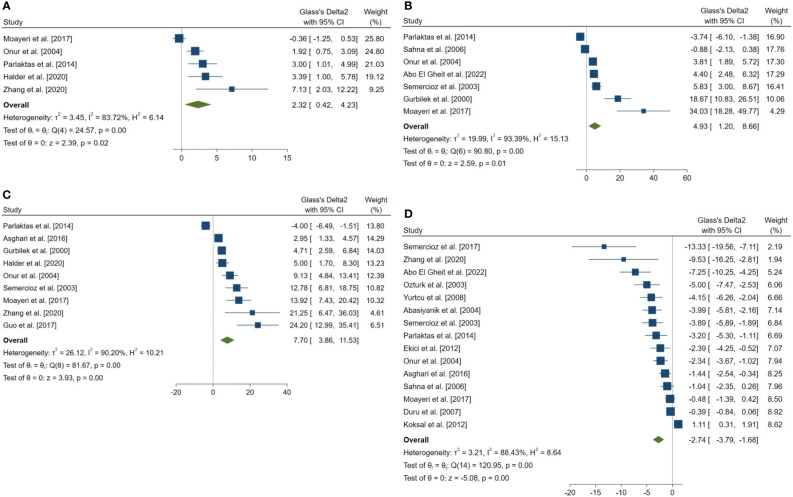
Forest plots for the overall pooled effects sizes of the testicular tissue oxidative stress markers. **(A)** CAT (n=5), **(B)** GPx (n=7), **(C)** SOD (n=9), **(D)** MDA (n=15). MDA, Malondialdehyde; GPx, Glutathione Peroxidase; CAT, Catalase; SOD, Superoxide Dismutase.

**Figure 6:**
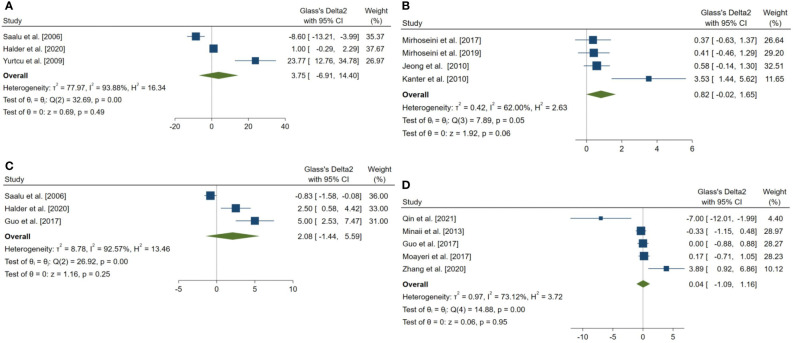
Forest plots for the overall pooled effects sizes of body weight and testicular somatic indices. **(A)** total testicular weight (n=3), **(B)** seminiferous tubular diameter (n=4), **(C)** final body weight (n=3), **(D)** testis to body relative weight (n=5).

**Figure 7:**
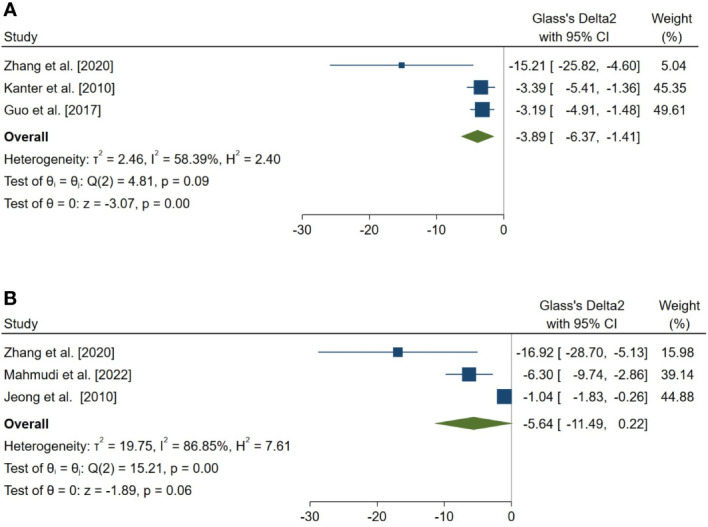
Forest plots for the overall pooled effects sizes of exploratory outcomes, including **(A)** the percentage of tubules with TUNEL-positive cells (n=3) and **(B)** the number of TUNEL-positive cells per tubule (n=3). TUNEL, Terminal deoxynucleotidyl transferase dUTP nick end labeling.

18 outcomes were pooled from the included studies which were classified into five groups: (a) sperm parameters, (b) reproductive hormone profile, (c) markers of oxidative stress in testicular tissue, (d) body weight and testicular somatic indices, and (e) exploratory outcomes. Pooled outcomes included: total sperm count, forward progressive motility, normal sperm morphology, sperm viability, Johnson tubular biopsy score, serum testosterone and Inhibin-B levels, testicular tissue SOD, malondialdehyde (MDA), GPx, and catalase (CAT) activity, final body and testes weight, testis to body weight ratio, seminiferous tubular diameter, percentage of tubules with TUNEL-positive cells (TUNEL: Terminal deoxynucleotidyl transferase dUTP nick end labeling), and number of TUNEL-positive cells per tubule. The forest plots of the overall pooled effects sizes are presented in the **Figure 3**–**7**.

#### 3.3.1 Sperm parameters

The combined SMDs for the effect of melatonin therapy on total sperm count (SMD = 2.358, 95% CI: 0.285 to 4.431, p-value = 0.026), forward progressive motility (SMD = 5.907, 95% CI: 4 to 7.814, p-value <0.001), normal sperm morphology (SMD = 3.312, 95% CI: 1.516 to 5.108, p-value <0.001), and sperm viability (SMD = 2.116, 95% CI: 0.291 to 3.941, p-value = 0.023) were statistically significant. On the other hand, total sperm motility was not significantly affected by melatonin therapy (SMD = -1.893, 95% CI: -9.076 to 5.29, p-value = 0.605). Between study heterogeneity was considerable for sperm viability (I^2^ = 82.5% and p-value for Q test <0.001), total sperm count (I^2^ = 87.55% and p-value for Q test <0.001), total sperm motility (I2 = 92.45% and p-value for Q test <0.001), and normal sperm morphology (I^2^ = 84.78% and p-value for Q test <0.001) and moderate for forward progressive sperm motility (I^2^ = 45.57% and p-value for Q test = 0.138).

Johnsen’s score was examined and reported in 14 studies, of which, 11 were included in the meta-analysis. The combined SMD for the effect of melatonin therapy on Johnsen’s mean testicular biopsy score was (SMD = 3.322, 95% CI: 1.759 to 4.885, p-value <0.001). Substantial between study heterogeneity was observed in the analysis.

#### 3.3.2 Reproductive hormone profile

The overall pooled SMDs for the effect of melatonin therapy on rodents’ reproductive hormones were (SMD = 1.012, 95% CI: -1.991 to 4.015, p-value = 0.509) and (SMD = 2.659, 95% CI: 1.296 to 4.022, p-value <0.001) for serum testosterone and Inhibin-B levels, respectively. Substantial between study heterogeneity was observed in both analyses (I^2^ = 89.03% and p-value for Q test <0.001 and I^2^ = 65.95% and p-value for Q test = 0.032 for serum testosterone and Inhibin-B levels, respectively).

#### 3.3.3 Markers of oxidative stress in testicular tissue

The pooled SMDs for the effect of melatonin therapy on testicular tissue antioxidant activity were for SOD (SMD = 7.698, 95% CI: 3.863 to 11.533, p-value <0.001), malondialdehyde (SMD = -2.738, 95% CI: -3.795 to -1.681, p-value <0.001), GPx (SMD = 4.927, 95% CI: 1.197 to 8.658, p-value = 0.005), and catalase (CAT, SMD = 2.323, 95% CI: 0.42 to 4.226, p-value = 0.017) were. However, considerable between-study heterogeneity (I^2^ = 90.2% and p-value for Q test <0.001 for SOD, I^2^ = 88.43% and p-value for Q test <0.001 for MDA, I^2^ = 93.39% and p-value for Q test <0.001 for GPx, and I^2^ = 83.72% and p-value for Q test <0.001 for CAT) was observed in testicular tissue antioxidant activities.

#### 3.3.4 Body weight and testicular somatic indices

The pooled SMDs for the effect of melatonin therapy on body weight and testicular somatic indices were not statistically significant; for final body weight (SMD = 2.076, 95% CI: -1.438 to 5.59, p-value = 0.247), final total testis weight (SMD = 3.745, 95% CI: -6.905 to 14.396, p-value = 0.491), testis to body weight ratio (SMD = 0.036, 95% CI: -1.089 to 1.162, p-value = 0.95), and seminiferous tubular diameter (SMD = 0.818, 95% CI: -0.018 to 1.655, p-value = 0.055). The between-study heterogeneity was substantial to considerable for body weight and testicular somatic indices; for final body weight (I^2^ = 92.57% and p-value for Q test <0.001), final total testis weight (I^2^ = 93.88% and p-value for Q test <0.001), testis to body weight ratio (I^2^ = 73.12% and p-value for Q test = 0.005), and seminiferous tubular diameter (I^2^ = 62% and p-value for Q test 0.048).

### 3.4 Exploratory outcomes

In a complementary analysis, we assessed the effect of melatonin therapy on TUNEL assay of seminiferous tubular cells (54, 65, 66, 68, 83, 85). The pooled SMD for the effect of melatonin therapy on the percentage of tubules with TUNEL-positive cells (SMD = -3.886, 95% CI: -6.365 to -1.406, p-value = 0.002) was statistically significant. On the other hand, this measure was not statistically significant for the number of TUNEL-positive cells per tubule (SMD = -5.636, 95% CI: -11.495 to 0.222, p-value = 0.059).

### 3.5 Publication bias

Two outcomes were eligible for analysis of publication bias. The funnel plots regarding the effects of melatonin therapy on testicular tissue MDA activity and Johnsen have been analyzed for publication bias. Both plots lack symmetry on visual inspection, suggesting a high risk of publication bias. Egger’s regression and Begg’s tests also showed consistent results: p-value <0.001 for both tests in both outcomes. The funnel plots are presented in **Supplementary Material 4**.

### 3.6 Subgroup analyses

Subgroup analyses were done on the side of induction of stress (unilateral vs. bilateral), mechanism of stress (ischemic vs. non-ischemic injuries), and duration of melatonin therapy (> 2 weeks vs. ≤ 2 weeks). Subgroup analyses revealed significant between-group differences for SOD based on the mechanism of stress and duration of melatonin therapy (p-value <0.001 and 0.01, respectively). All the meta-analyses are stratified in **Table 2**.

**Table 2:** Subgroup analyses.

Outcome		Subgroup	Number of effect sizes	Pooled SMD [95% CI]	P-for-difference	P-for-heterogeneity	I^2^ (%)
**GPx**	All		7	4.927 [1.197, 8.658]	0.005	<0.001	93.39
	Mechanism of stress	Ischemia-induced stress	3	2.650 [-3.300, 8.610]	0.370	<0.001	93.18
		Non-ischemia-induced stress	4	5.750 [2.510, 8.980]		<0.001	79.79
	Treatment duration	More than 2 weeks	4	5.748 [2.514, 8.983]	0.370	<0.001	79.79
		2 weeks and less	3	2.652 [-3.303, 8.607]		<0.001	93.18
**Johnsen score**	All		11	3.322 [1.759, 4.885]	<0.001	<0.001	87.47
	Mechanism of stress	Ischemia-induced stress	8	3.694 [1.804, 5.585]	0.59	<0.001	84.84
		Non-ischemia-induced stress	3	2.660 [-0.620, 5.930]		<0.001	91.46
**MDA**	All		15	-2.738 [-3.795, -1.681]	<0.001	<0.001	88.43
	Mechanism of stress	Ischemia-induced stress	10	-2.408 [-3.665, -1.151]	0.33	<0.001	89.09
		Non-ischemia-induced stress	5	-3.73 [-6.070, -1.380]		<0.001	87.00
	Treatment duration	More than 2 weeks	5	-4.297 [-6.897, -1.697]	0.16	<0.001	89.71
		2 weeks and less	10	-2.224 [-3.417, -1.030]		<0.001	87.72
**SOD**	All		9	7.698 [3.863, 11.533]	<0.001	<0.001	90.2
	Mechanism of stress	Ischemia-induced stress	3	1.286 [-3.299, 5.870]	<0.001	<0.001	93.29
		Non-ischemia-induced stress	6	12.31 [7.390, 17.240]		<0.001	73.78
	Treatment duration	More than 2 weeks	5	11.495 [6.524, 16.467]	0.014	0.002	75.69
		2 weeks and less	4	2.848 [-1.909, 7.605]		<0.001	91.74
	Stress side	Bilateral	4	14.660 [5.528, 23.791]	0.05	0.001	82.75
		Unilateral	5	4.575 [0.270, 8.879]		<0.001	92.13

GPx, glutathione peroxidase; MDA, malondialdehyde; SOD, superoxide dismutase.

### 3.7 Risk of bias assessment

**Figure 8:**
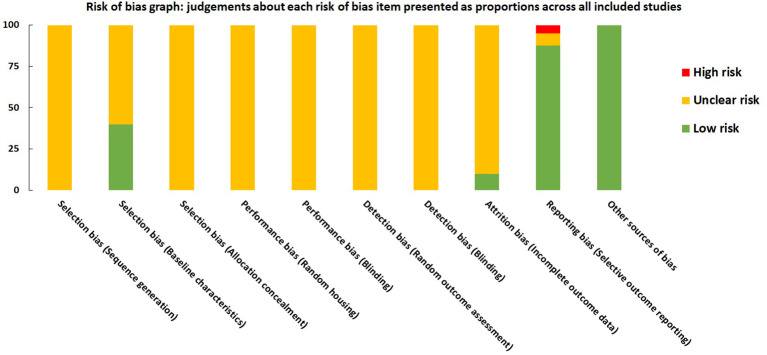
Risk of bias graph on judgements about each risk of bias item presented as proportions across all included studies (n=41).

For each domain, studies scored 1 if they were assessed as low risk using SYRCLE tool. Studies scored between 1 and 4. All the studies were labeled as unclear risk on random sequence generation, allocation concealment, random housing, blinding, and random outcome assessment. For other sources of bias, all the studies were assessed as low risk. Studies were low risk based on baseline characteristics (n=16), incomplete outcome data (n=4), and selective outcome reporting (n= 35). All the details are presented in **Figure 8** and **Supplementary Material 5**.

## 4 Discussion

Our results suggest the beneficial effects of melatonin on male rodents’ infertility caused by physical testicular injuries. We hypothesized that melatonin might exert this favorable effect by influencing the reproductive system, inducing antioxidant defense, and suppressing apoptosis. Furthermore, subgroup analysis revealed a stronger impact of melatonin on testicular SOD in non-ischemia-induced injuries and studies with longer intervention duration compared to the comparison groups. Treatment duration and mechanism of stress were detected as possible sources of heterogeneity for testicular SOD and GPx analysis. In the following, we discussed the possible related mechanisms.

### 4.1 Effects on sperm and testis parameters

Male infertility is commonly caused by ejaculatory dysfunction, no or low sperm count, or abnormal morphology or motility of the sperms (86). In the present study, melatonin improved spermatogenesis, total sperm count, sperm viability and morphology, and forward progression sperm motility in animals with infertility induced by physical injuries. However, total testicular weight, testis to body weight ratio, sperm motility, and seminiferous tubule diameter were not affected by melatonin administration. Our results were partially in line with previous studies on other models of male infertility. Melatonin protects the reproductive system against the toxicity of chemotherapeutic agents (87). Zi et al. reported the favorable effects of melatonin on doxorubicin-induced impaired spermatogenesis and sperm quality, except for sperm motility (88). Melatonin also suppressed bleomycin, etoposide, and cisplatin-induced testicular damage by ameliorating histopathological alterations of testes, testicular weight, and sperm motility, viability, and morphology, but not sperm count and its progressive motility (89). These discrepancies may be due to the differences in the type of animals, experimental models of male infertility, and dose, duration, and route of melatonin administration. Melatonin may directly affect the male reproductive system by interacting with its receptors on the testes, epididymis, or spermatozoa (90, 91). Melatonin increased the expression of spermatogenesis-related genes and led bovine Sertoli cells to transit from G1 to S phase (92). In addition to the experimental studies, lower serum and seminal melatonin levels in patients with idiopathic oligoasthenoteratozoospermia compared to the fertile men and a significant positive correlation between serum melatonin and sperm motility were detected in a previous case-control study (93). Due to the conflicting evidence (55, 91), more studies should be performed to clarify the effects of melatonin on sperm and testis parameters.

### 4.2 Effects on reproductive hormone levels

Testosterone has fundamental roles in male reproductive system development and spermatogenesis (94). Our results revealed that melatonin does not affect plasma testosterone levels, similar to some reports on other animal models of male infertility (89, 95). Nonetheless, transgenic mammals with endogenously elevated melatonin levels revealed elevated testosterone production (96). Evidence also suggests that melatonin induces testosterone activation and inhibits its destruction (97). Although, the low number of included studies may conceal the possible influence of melatonin on testosterone; consequently, further studies are needed to address the issue.

The serum level of inhibin-B, a hormone produced primarily by the Sertoli cells of the testes, is a potential marker of testicular function and spermatogenesis (98, 99). Our findings demonstrated the improvement of inhibin-B levels following melatonin administration, which may be because of melatonin’s beneficial effects on preserving Sertoli cells against injuries. In this regard, melatonin upregulated inhibin-B expression in bovine Sertoli cells *via* its MT1 and MT2 receptors (92). In a randomized controlled trial by Lu et al., melatonin supplementation increased the peripheral blood inhibin B levels following varicocelectomy that could be attributed to its effects on spermatogenesis function (100).

### 4.3 Effects on oxidant/antioxidant balance

Testicular oxidative stress could be derived from intrinsic etiologies, including testicular torsion, varicocele, cryptorchidism, infection, inflammation, or aging (101). Some extrinsic factors, such as intense exercise, can also raise oxidative stress levels in the testes (69). Sperms are extremely sensitive to oxidative damage due to the high levels of polyunsaturated fatty acids in their membranes and the low content of enzymatic antioxidants. Oxidative stress is directly correlated with increased apoptosis in germ cells and mature spermatozoa by changing caspase activity and disrupting mitochondrial membrane (101). Therefore, multiple studies indicated the association between oxidative stress and abnormal sperm count, motility, viability, morphology, DNA integrity, and fertilization ability (5, 101, 102). According to our results, melatonin administration significantly increased the SOD, GPx, and CAT activities, essential enzymatic antioxidants, and reduced MDA levels, as a lipid peroxidation product, in the testicular tissue of animals. Antioxidant properties of melatonin are reported in previous literature. Melatonin could directly scavenge oxidants and indirectly increase enzymatic antioxidant levels (103). Morvaridzadeh et al. conducted a meta-analysis of randomized controlled trials on patients with a different health condition. They reported increased total antioxidant capacity, glutathione levels, SOD, GPx, and glutathione reductase, but not CAT activities, and reduced MDA levels following melatonin supplementation (104). Melatonin could upregulate the antioxidant nuclear factor erythroid 2-related factor 2 (Nrf2)/heme oxygenase-1 (HO-1) signaling pathway in the damaged testes (83). This pathway induces the transcription of antioxidant proteins and leads to ROS clearance (105). Melatonin may also exert its beneficial effects through upregulating micro-RNA-34a/silent information regulator 1 (SIRT1)/forkhead transcription factors-class O (type1) (FOXO1) epigenetic axis (60). This pathway stimulates antioxidants’ expression, inhibits pro-inflammatory pathways, decreases apoptosis, improves mitochondrial biogenesis, repairs cell damage, and prevents cells’ dysfunction and infertility (106). Therefore, the effects of melatonin on other oxidative stress-related conditions should be investigated.

### 4.4 Effects on apoptosis

Apoptosis, the programmed cell death, takes place during normal spermatogenesis. However, physical testicular injuries could elevate germ cell apoptosis and reduce seminiferous tubule diameter and sperm count (60, 68, 107, 108). We observed that melatonin decreases apoptotic germ cells and ameliorates the detrimental impact of physical injuries on the testes. Consistently, melatonin had protective effects against apoptotic cell damage caused by radiation (109, 110) or drugs (89) in the testes in other studies. Melatonin also inhibited endoplasmic reticulum stress-induced apoptosis in reproductive tissues, and therefore exerted protective effects on diabetes-related reproductive impairment (111). The anti-apoptotic effect of melatonin could be mediated *via* increasing Bcl-2 gene expression, an anti-apoptotic gene marker, and lowering mitochondrial membrane potentials and pro-apoptotic Bcl-2-associated X protein (BAX), P53, caspase 3, and nuclear factor-κB (NF-κB) expression (83, 89, 112, 113). Melatonin also activated the phosphoinositide 3-kinase (PI3K)/protein kinase B (AKT) pathway in frozen-thawed human sperms (114). Activation of this pathway causes increased sperm motility, suppressed apoptotic cascade and caspase, and decreased membrane permeability and ROS production in spermatozoa (115). Furthermore, melatonin protected human spermatozoa from H_2_O_2_-induced DNA fragmentation and apoptosis *via* MT1 and extracellular signal–regulated kinase signaling (116). Some other studies also support the anti-apoptotic effects of melatonin on injuries to the female reproductive system (117, 118), heart (113), kidney (119), liver (120), and nervous tissue (121). Due to the limited evidence, we recommend that future studies assess the mechanisms related to the anti-apoptotic effects of melatonin in physical injuries to male testicular tissue.

### 4.5 Strengths and limitations

To the best of our knowledge, this is the first systematic review and meta-analysis on the protective effects of melatonin against physical injuries to rodents’ testicular tissue. Animal models are indispensable tools for assessing new agents’ effectiveness and side effects for disease management. However, they do not completely imitate human models. Therefore, the interpretation of our findings should be conducted with caution. High statistical heterogeneity, publication bias, and low quality of the eligible studies are other limitations of our meta-analysis. Between-study methodological heterogeneity was also found in our study due to the differences between animals’ characteristics, the dose of melatonin, the intervention schedule, and model of infertility induction. Furthermore, the low number of the included studies prohibited us from doing subgroup analysis for some variables to detect other sources of heterogeneity. Almost all the available studies that have utilized animal models to evaluate the effects of melatonin therapy against male infertility were on rodent subjects. None of the included studies have evaluated and reported possible adverse effects of melatonin therapy. Finally, there are other outcomes that could have been helpful in explaining the mechanisms behind the effects of exogenous melatonin on male rodents’ reproductive system such as dihydrotestosterone, corticosterone, testicular and general immunity which were not investigated by the included studies.

## 5 Conclusion and future direction

In conclusion, melatonin protects against male infertility caused by physical injuries through direct effects on rodent male reproductive system cells, inducing antioxidant defense, and inhibiting apoptosis. More well-designed animal studies should be performed to clarify other mechanisms underlying these effects. To avoid methodological variations, future studies should be more harmonized regarding the mechanism of injury and design of treatment to develop consensus on definitions and methods in this field of research. Also, we recommend employing non-rodent subjects to assess generalizability of the results of this review. Discussions should be made about concerns on melatonin therapy with doses that are needed for anti-infertility effects. Future studies should consider detecting possible adverse effects and other outcomes such as dihydrotestosterone, corticosterone in their protocols.

## Data availability statement

The original contributions presented in the study are included in the article/**Supplementary Material**. Further inquiries can be directed to the corresponding author.

## Author contributions

NA and NDE conceptualized the study. AS, NDE, SS-Z, and ARS designed the study. NDE, AS, and SS-Z searched databases. NDE, NM, and SP screened the records. NDE, ET, FSS, and AS extracted the data. NDE and AS performed quality assessment. AS, SS-Z, and ARS performed meta-analysis. AS, SS-Z, SD, NH, and ZM provided the draft of the manuscript. AS visualized the data. NA and ARS supervised the work. All authors contributed to the article and approved the final version. AS and NDE have contributed equally to this work and share first authorship. All authors contributed to the article and approved the submitted version.
