# Supplementary material for: Protective effects of melatonin against physical injuries to testicular tissue: A systematic review and meta-analysis of animal models
Source: Front Endocrinol (Lausanne). 2023 Jan 31;14:1123999. doi: 10.3389/fendo.2023.1123999 (PMC9927015; doi:10.3389/fendo.2023.1123999)
Supplement: Supplementary file 1 [file Table_1.docx]

# Supplementary material 1

**Search strategy**

| **Keywords** | | | |
| --- | --- | --- | --- |
| Melatonin | Testicular function | | Mesh terms |
| Melatonin | "sertoli Cell" | Sterility | Infertility |
| “N-acetyl-5-methoxy tryptamine” | seminal | Subfertility | Fertility |
| “n acetyl 5 methoxytryptamine” | fertil* | “Sub-Fertility” | Fertility Agents, Male |
| "Mela-T" | epididymis | Aspermia | Testis |
| Melatol | "Vas deferens" | Fecundability | Epididymis |
| Melatonex | testes | Fecundity | Vas deferens |
| Melovine | testis | Subfecundity | Spermatogenesis |
| Regulin | testicular | Testicles | Testosterone |
| “Night NEXT Rest” | sterility | Testicle | Seminiferous Tubules |
| Circadin | sperm | “Ductus Deferens” | Seminiferous Epithelium |
|  | spermat* | Fertility AND Agents AND Male | Spermatozoa |
|  | Semen | Spermiogenesis | Semen Analysis |
|  | reproduction | Semen AND Analysis | Genitalia, Male |
|  | preconception | “17-beta-Hydroxy-4-Androsten-3-one” | Melatonin |
|  | testosterone | “17 beta Hydroxy 4 Androsten 3 one” |  |
|  | "leydig cell" | "Seminiferous Tubule" |  |
|  | seminiferous | "Seminiferous Tubules" |  |
|  | oligospermia | Seminiferous AND epithelium |  |
|  | azoospermia | Seminiferous AND epitheliums |  |
|  | astenozoospermia | Semen AND Analyses |  |
|  | infertil* | Semen AND Quality |  |
|  | Hypospermatogenes* | Sperm AND Quality |  |
|  | Oligoasthenoteratozoospermia* | Semen AND Qualities |  |
|  | Oligozoospermia | Sperm AND Qualities |  |
|  | Genital* AND Male |  |  |
|  | Reproductive AND Male |  |  |

## PubMed

(Melatonin[tiab] OR "N-acetyl-5-methoxy tryptamine"[tiab] OR "n acetyl 5 methoxytryptamine"[tiab] OR "Mela-T"[tiab] OR Melatol[tiab] OR Melatonex[tiab] OR Melovine[tiab] OR Regulin[tiab] OR "Night NEXT Rest"[tiab] OR Circadin[tiab]) AND ("sertoli Cell"[tiab] OR (sertoli[tiab] AND cell*[tiab]) OR seminal[tiab] OR fertil*[tiab] OR epididymis[tiab] OR "vas deferens"[tiab] OR (vas[tiab] AND deferens[tiab]) OR testes[tiab] OR testis[tiab] OR testicular [tiab] OR sterility[tiab] OR sperm*[tiab] OR Semen[tiab] OR reproduction[tiab] OR preconception[tiab] OR testosterone[tiab] OR "leydig cell"[tiab] OR (leydig[tiab] AND cell*[tiab]) OR "Seminiferous Tubule"[tiab] OR "Seminiferous epithelium"[tiab] OR (Seminiferous[tiab] AND Tubule*[tiab]) OR (Seminiferous[tiab] AND epithelium*[tiab]) OR oligospermia[tiab] OR hypospermatogenes*[tiab] OR Oligoasthenoteratozoospermia*[tiab] OR Oligozoospermia[tiab] OR azoospermia[tiab] OR astenozoospermia[tiab] OR infertil*[tiab] OR subfertility[tiab] OR "Sub-fertility"[tiab] OR fertil*[tiab] OR fecundability[tiab] OR fecundity[tiab] OR subfecundity[tiab] OR Aspermia[tiab] OR testicle*[tiab] OR (Ductus[tiab] AND Deferens[tiab]) OR "Ductus Deferens"[tiab] OR ("male fertility"[tiab] AND Agents[tiab]) OR "17-beta-Hydroxy-4-Androsten-3-one"[tiab] OR "17 beta Hydroxy 4 Androsten 3 one"[tiab] OR (semen[tiab] AND analys*[tiab]) OR (semen[tiab] AND qualit*[tiab]) OR (sperm[tiab] AND qualit*[tiab]) OR (Genital*[tiab] AND Male[tiab]) OR (Reproducti*[tiab] AND Male[tiab])) OR ((Melatonin[Mesh]) AND ((“Infertility”[Mesh]) OR (“Fertility”[Mesh]) OR (“Fertility Agents, Male”[Mesh]) OR (“Testis”[Mesh]) OR (“Epididymis”[Mesh]) OR (“Vas deferens”[Mesh]) OR (“Spermatogenesis”[Mesh]) OR (“Testosterone”[Mesh]) OR (“Seminiferous Tubules”[Mesh]) OR (“Seminiferous Epithelium”[Mesh]) OR (“Spermatozoa”[Mesh]) OR (“Semen Analysis”[Mesh]) OR (“Genitalia, Male”[Mesh])) AND (1965/1/1:2022/9/9[dp])

NNR: 12.5

**Results: 1375**

Date of search: September 9, 2022

## Scopus

TITLE-ABS-KEY(Melatonin OR "N-acetyl-5-methoxy tryptamine" OR "n acetyl 5 methoxytryptamine" OR "Mela-T" OR Melatol OR Melatonex OR Melovine OR Regulin OR "Night NEXT Rest" OR Circadin) AND TITLE-ABS-KEY("sertoli Cell" OR (sertoli AND cell*) OR seminal OR fertil* OR epididymis OR "vas deferens" OR (vas AND deferens) OR testes OR testis OR testicular OR sterility OR sperm* OR Semen OR reproduction OR preconception OR testosterone OR "leydig cell" OR (leydig AND cell*) OR "Seminiferous Tubule" OR "Seminiferous epithelium" OR (Seminiferous AND Tubule*) OR (Seminiferous AND epithelium*) OR oligospermia OR hypospermatogenes* OR Oligoasthenoteratozoospermia* OR Oligozoospermia OR azoospermia OR astenozoospermia OR infertil* OR subfertility OR "Sub-fertility" OR fertil* OR fecundability OR fecundity OR subfecundity OR Aspermia OR testicle* OR (Ductus AND Deferens) OR "Ductus Deferens" OR ("male fertility" AND Agents) OR "17-beta-Hydroxy-4-Androsten-3-one" OR "17 beta Hydroxy 4 Androsten 3 one" OR (semen AND analys*) OR (semen AND qualit*) OR (sperm AND qualit*) OR (Genital* AND Male) OR (Reproducti* AND Male)) AND PUBYEAR > 1965 AND PUBYEAR < 2023

**Results: 4826**

Date of search: September 9, 2022

## Web of science

TS=(Melatonin OR "N-acetyl-5-methoxy tryptamine" OR "n acetyl 5 methoxytryptamine" OR "Mela-T" OR Melatol OR Melatonex OR Melovine OR Regulin OR "Night NEXT Rest" OR Circadin) AND TS=("sertoli Cell" OR (sertoli AND cell*) OR seminal OR fertil* OR epididymis OR "vas deferens" OR (vas AND deferens) OR testes OR testis OR testicular OR sterility OR sperm* OR Semen OR reproduction OR preconception OR testosterone OR "leydig cell" OR (leydig AND cell*) OR "Seminiferous Tubule" OR "Seminiferous epithelium" OR (Seminiferous AND Tubule*) OR (Seminiferous AND epithelium*) OR oligospermia OR hypospermatogenes* OR Oligoasthenoteratozoospermia* OR Oligozoospermia OR azoospermia OR astenozoospermia OR infertil* OR subfertility OR "Sub-fertility" OR fertil* OR fecundability OR fecundity OR subfecundity OR Aspermia OR testicle* OR (Ductus AND Deferens) OR "Ductus Deferens" OR ("male fertility" AND Agents) OR "17-beta-Hydroxy-4-Androsten-3-one" OR "17 beta Hydroxy 4 Androsten 3 one" OR (semen AND analys*) OR (semen AND qualit*) OR (sperm AND qualit*) OR (Genital* AND Male) OR (Reproducti* AND Male)) AND DOP=(1965-01-01/2022-09-09)

**Results: 3838**

# Supplementary material 2

| Additional information on the studies including detailed injury mechanism, timing and duration of melatonin therapy, and timing of assessment for outcomes. N/M, not mentioned. | | | |
| --- | --- | --- | --- |
| First author [year] | Injury mechanism | Timing and duration of melatonin therapy | Timing of assessment |
| Abasiyanik [2004] (1) | 720° clockwise torsion for 6 hours and then detorsion | During injury (15 minutes before detorsion) | 6 hours after detorsion |
| Abo El Gheit [2021] (2) | Partial ligation of renal vein | For 4 weeks, started one week after injury induction | 5 weeks later |
| Aktas [2011] (3) | 720° torsion for 2.5 hours and then detorsion | After detorsion | N/M |
| Asghari [2016] (4) | Clamping for 1 hours | For one week after injury induction | 7 days after injury induction |
| Bustos-Obregón [2010] & Hartley [2009] (5, 6) | 1-5 cycles of hypoxia-normoxia | 7 to 70 days | 1, 2, 4, 6, 8, and 10 weeks after study start |
| Chen [2021] (7) | 720° counterclockwise torsion for 2 hours and then detorsion | Once during injury (30 minutes after torsion), and then at 3 hours, one day, 2 days and 3 days after injury induction | 3 days later |
| Duru [2007] (8) | 720° counterclockwise torsion for 1, 3, and 5 hours and then detorsion | One arm before injury induction (before torsion) and one arm during injury induction (before detorsion) | 1.5 hours after detorsion |
| Ekici [2012] (9) | 720° clockwise torsion for 6 hours and then detorsion | First during injury induction (15 minutes before detorsion) and then daily for a week | 7 days later |
| Erdemir [2008] (10) | 720° clockwise torsion for 2 hours and then detorsion | During injury (30 minutes before detorsion) | 2 hours after detorsion |
| Esrefoglu [2004] (11) | To provide arterial occlusion, the ligature was tightened for 30 minutes, and reperfusion was achieved by releasing the tension. | 10 minutes before & during ischemia | Immediately after 2 hours reperfusion |
| Gul [2018] (12) | Left testis: 720° clockwise torsion for 2 hours and then detorsion | One arm during injury (single dose 30 minutes before detorsion) and one arm for one week | 2 hours and 1 week later |
| Guo [2017] (13) | 6 hours/day of immobilization stress in 50 mL conical centrifuge tubes for 35 days | 35 days | 35 days later |
| Gürbilek [2000] (14) | Left testis: 720° clockwise torsion for 6 hours and then detorsion | During injury (single dose 15 minutes before detorsion till 1 hour afterwards) | 6 hours later |
| Haldera [2020] (15) | Hyperthermia induction chamber | 15 days | N/M |
| Jeong [2010] (16) | 720° counterclockwise torsion for 4 hours and then detorsion | During injury (15 minutes before detorsion) | 3 and 7 weeks later |
| Kanter [2010] (17) | 720° clockwise torsion for 5 hours and then detorsion | During injury (40 minutes before detorsion) | 5 hours later |
| Koksal [2012] (18) | Clamping for 1 hours | 10 minutes before injury | 24 hours later |
| Kurcer [2008] (19) | Clamping for 1 hours | 10 minutes before injury | Histologic assessment: 24 hours later and sperm profile analysis: 30 days later |
| Kurcer [2010] (20) | Clamping for 1 hours | During the injury (10 minutes before removal of clamping and 1 hour afterwards) | Histologic assessment: 24 hours later and sperm profile analysis: 30 days later |
| Mahmudi [2022] (21) | 1 hour of forced treadmill exercise daily, 5 days a week for 8 weeks | 8 weeks meanwhile with injury induction | N/M |
| Minaii [2013] (22) | 1 hour of continuous swimming daily, 5 days a week for 8 weeks | 8 weeks meanwhile with injury induction | N/M |
| Mirhoseini [2017] (23) | 720° clockwise torsion for 1 hour and then detorsion | Just after detorsion | 72 hours later |
| Mirhoseini [2019] (24) | After firming animals and their testis on a plate, the testicle was dropped by 25 g sinker for 4 times | Just after trauma | 72 hours later |
| Moayeri [2017] (25) | 1 hour of continuous swimming daily, 5 days a week for 8 weeks | 8 weeks meanwhile with injury induction | N/M |
| Olayaki [2017] (26) | N/M | For 30 days after injury | 30 days later |
| Onur [2004] (27) | Partial ligation of renal vein | For 30 days after injury | 30 days later |
| Ozturk [2003] (28) | 720° torsion for 6 hours and then detorsion | During the injury before detorsion | 12 hours after detorsion |
| Parlaktas [2014] (29) | 720° clockwise torsion for 2 hours and then detorsion | During injury (30 minutes before detorsion) | 2 hours after detorsion |
| Qin [2021] (30) | Lower body part in a 42 °C thermostatic water bath for 20 minutes | Over 1 week before injury | 12 hours after injury induction |
| Saalu [2006] (31) | Anchoring the upper pole of testis to the abdominal wall | For 56 days after injury induction | After 56 days of melatonin administration |
| Sahna [2006] (32) | A branch of the descending left coronary artery was occluded for 30 minutes, followed by 120-minute reperfusion | 10 minutes before ischemia | As soon as reperfusion was completed |
| Sekmenli [2016] (33) | 720° clockwise torsion for 6 hours and then detorsion (7 days of reperfusion) | Once 15 minutes before injury and then daily for one week | 7 days later |
| Semercioz [2003] (34) | Partial ligation of renal vein | Over 4 weeks after the injury | 4 weeks after injury induction |
| Semercioz [2017] (35) | 720° clockwise torsion for 1 hour and then detorsion | Over 3 weeks prior to injury | 1 hour after detorsion |
| Vargas [2011] (36) | Simulated hypoxia of 4,200 m above sea level in a chamber for 33.2 days ± an intermittency period of 4 days hypoxia /4 days normoxia | During the 33.2 days of injury induction | After the 33.2 days of injury induction |
| Yildirim [2006] (37) | 720° torsion for 2.5 hour and then detorsion | During injury | 2.5 hours after detorsion |
| Yuan [2016] (38) | Modified weight-drop model (an impact of 50 g/mm to the dorsal surface of the spinal cord) | 30 minutes after injury | N/M |
| Yurtçu [2008] (39) | 720° clockwise torsion for 6 hours and then detorsion | Once 15 minutes before injury and then daily for one week | 7 days later |
| Yurtçu [2009] (40) | 720° clockwise torsion for 6 hours and then detorsion | One arm 15 minutes before injury and one arm once 15 minutes before injury and then daily for one week | 6 hours after detorsion |
| Zhang [2020] (41) | Lower body part in a 42 °C thermostatic water bath for 20 minutes | One arm 2 hours before injury and one arm for 2 weeks after injury | 6 and 12 hours, 7, 14, 21 and 35 days after injury |

# Supplementary material 3

| Descriptive results of histopathological findings. N/M, not mentioned. | | |
| --- | --- | --- |
| First author [year] | **Electron microscopy** | |
|  | *Interventions* | *Controls* |
| Yildirim [2006] (37) | Slight lipid accumulation in the basal cells cytoplasm, slightly more lipid accumulation in the columnar cells, mitochondrial crystolysis with more myelin figures due to the cytoplasm edema | Increased lipid contents in the basal cells, electron dense lipid accumulation, more autophagic vacuoles, increased seconder lysosomes, mitochondrial crystolysis in columnar cells, some area of columnar cells necrosis and extensive degeneration |
| Kanter [2010] (17) | Melatonin treatment effectively prevented the mitochondrial degeneration, dilatation of SER, and intercellular spaces | Dilated cisternae of SER, swollen mitochondria with degenerated cristae and enlarged intercellular spaces in both Sertoli and spermatid cells |
| Aktas [2011] (3) | Sertoli and spermatogonia cells showing membrane-like structures and cytoplasmic voids. Small mitochondrions and vacuole-like structures placed on the edge. With increased melatonin dose more similar to normal testicular appearance. | Dissociations in spermatocide nuclei, many vacuoles and residual particles resulting from organelle degeneration/ Local voids in spermatogonia cytoplasms and dilatation in granulated endoplasmic reticulum seen in some sections/ Also, large lipid droplets, chromatid particles, along with mitochondrial crystalisis in some sections |
|  | **Light microscopy** | |
| Qin [2021] (30) | Numerous tubules were seen with normal seminiferous epithelium and vacuolization was rarely observed | Clumps of cells in the lumen and vacuolization in the tubules |
| Sekmenli [2016] (33) | Few spermatocytes with ischemic necrosis | Extensive necrosis without any visible spermatogenesis |
| Semercioz [2003] (34) | Partial preservation of normal histopathology; basal layer thickening with slightly decreased seminiferous tubule diameter and some loss of germ cells | Severe degenerative changes of the germinal epithelium and atrophy of the seminiferous tubules |
| Semercioz [2017] (35) | Spermatogenic activity is close to normal in much tubules | Cells are viewing as silhouette. Spermatogenic activity is lost much tubules and common necrosis |
| Yurtçu [2008] (39) | N/M | Atrophic testicular tissue |
| Yurtçu [2009] (40) | Atrophic testicular tissue, no seminiferous epithelial cells and tubular sclerosis | Atrophic testicular tissue |
| Jeong [2010] (16) | N/M | Interstitial edema, dilatation and germinal cell sloughing |
| Kanter [2010] (17) | Improved histological appearance than control group | Marked decrease in the seminiferous tubular diameter, with severe distortion, extensive disorganization, sloughing, and a loss of maturation of germ cells in tubules |
| Koksal [2012] (18) | Improving disorganization, increasing maturation and germ cell count | Extensive disorganization, sloughing and loss of maturation of germ cells |
| Kurcer [2010] (20) | Maturing spermatids and sloughed germ cells in the lumen | Disorganization and sloughing of germ cells into the tubule lumen |
| Kurcer [2008] (19) | Confined disorganization and sloughing epithelial cells only to a few tubules | Extensive disorganization, sloughing, and loss of maturation of germ cells |
| Mirhoseini [2017] (23) | Fewer tubules with detachment, vacuolization or some degrees of degeneration compared to the control | Extensive degenerative changes, as well as detachment and vacuolization/ Nearly all lineage of spermatogonial cells are damaged; there is no spermatid or spermatozoid in the lumen. |
| Mirhoseini [2019] (24) | N/M | Hematoma formation, detached seminiferous tubules, destroyed interstitial cells and increased distance between the tubules Some degrees of germ cell detachment and sloughing, and vacuolization, karyolitic cells between Sertoli cells |
| Asghari [2016] (4) | Normality of most seminiferous tubules | Markedly increase disorganization, sloughing and loss of maturation of germ cells/ seminiferous tubular degeneration, reduction of germ cell layer number, and spermatogenous arrest |
| Ekici [2012] (9) | Tubules showing germ cell necrosis besides the most tubules and incomplete maturation arrest up to the level of primary and secondary spermatocyte, with significant rescue of testicular function | Evidence of marked congestion with the presence of interstitial edema, PMN infiltration, and areas of focal hemorrhage/ Almost all of the tubules showing either only Sertoli cells without germinal cells or germ cell necrosis |
| Abo El Gheit [2021] (2) | Mostly intact spermatogenic cells with restored their orderly fashion with presence of sperms in the lumen, yet focal separation of spermatogenic cells was observed in some parts of the seminiferous tubules | Widely separated disorganized seminiferous tubules with dilated congested blood vessels in the interstitial spaces/ Mostly sloughed spermatogenic cells tubular basement membrane with signs of degeneration/ Necrotic cells in the lumen with absence of sperms |
| Esrefoglu [2004] (11) | Generally normal testicular architecture. | Tubular atrophy, hyalinization, degeneration, and disorganization of seminiferous epithelium. The lumina of the atrophic tubules contained occasional degenerated Sertoli cells. Numerous damaged tubules containing many degenerating cells with pyknotic or fragmented nuclei and acidophilic cytoplasm were observed. |
| Guo [2017] (13) | Unremarkable | Unremarkable |
| Haldera [2020] (15) | No significant degenerative changes in the seminiferous tubules. | Germ cell loss. Lack of spermocytes, round spermatids, and spermatozoa. Breakdown of seminiferous epithelium due to destruction of germinal epithelium, germ cells, spermocytes, and round spermatids. |

# Supplementary material 4

| 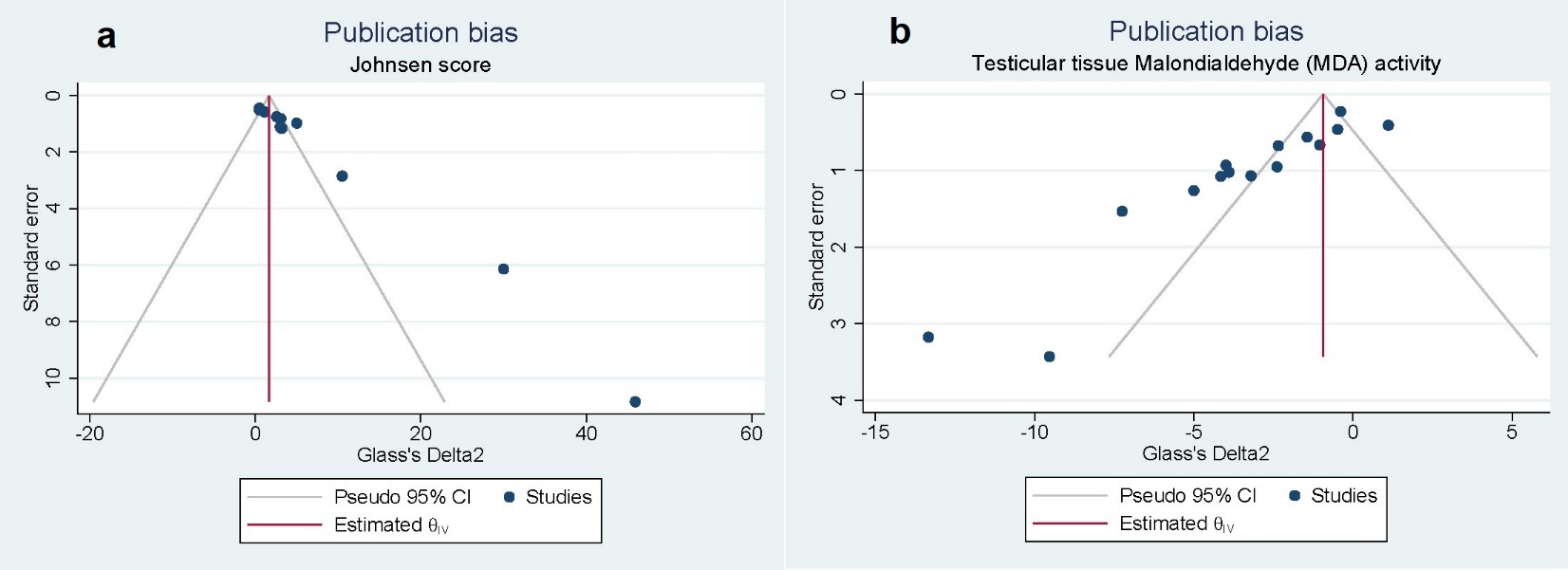 |
| --- |
| **Funnel plots for visual assessment of publication bias in Johnsen score and MDA. MDA, Malondialdehyde.** |

# Supplementary material 5

| 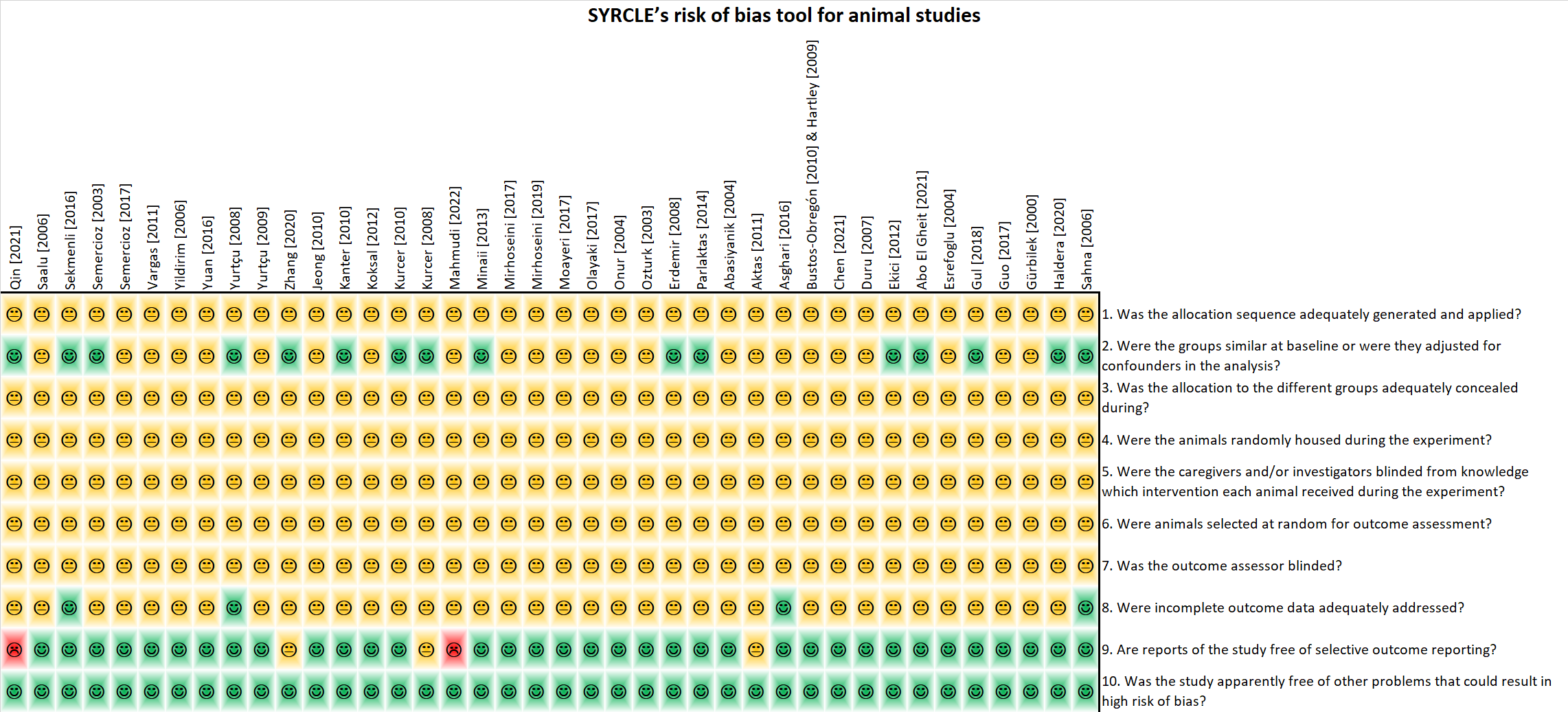 |
| --- |
| **Detailed judgement on risk of bias assessment for each study-item separately.** |

1. Abasiyanik A, Dağdönderen L. Beneficial effects of melatonin compared with allopurinol in experimental testicular torsion. Journal of Pediatric Surgery. 2004;39(8):1238-41.

2. Abo El Gheit RE, Soliman NA, Nagla SA, El-Sayed RM, Badawi GA, Emam MN, et al. Melatonin epigenetic potential on testicular functions and fertility profile in varicocele rat model is mediated by silent information regulator 1. Br J Pharmacol. 2022;179(13):3363-81.

3. Aktaş A, Tuncer M, Yıldırım A, Nergiz Y, Akkus M. Protective Effects of Melatonin on Testicular Torsion and Detorsion Damage in Sprague-Dawley Rats. International Journal of Morphology. 2011;29:7-15.

4. Asghari A, Akbari G, Meghdadi A, Mortazavi P. Effects of melatonin and metformin co-administration on testicular ischemia/reperfusion injury in rats. J Pediatr Urol. 2016;12(6):410.e1-.e7.

5. Bustos-Obregon E, Sánchez R, Ramos B, Torres-Diaz L. Rat Spermatogenesis Damage in Intermittent Hypobaric Hypoxia and the Protective Role of Melatonin. II: Testicular Parameters. International Journal of Morphology. 2010;28:537-47.

6. Hartley R, Castro-Sánchez R, Ramos-Gonzalez B, Bustos-Obregón E. Rat Spermatogenesis Damage in Intermittent Hypobaric Hypoxia and the Protective Role of Melatonin: I Cauda Epididymal Spermatozoa. International Journal of Morphology. 2009;27:1275-84.

7. Chen YT, Chuang FC, Yang CC, Chiang JY, Sung PH, Chu YC, et al. Combined melatonin-adipose derived mesenchymal stem cells therapy effectively protected the testis from testicular torsion-induced ischemia-reperfusion injury. Stem Cell Res Ther. 2021;12(1):370.

8. Duru FI, Noronha CC, Akinwande AI, Okanlawon AO. Effects of torsion, detorsion and melatonin on testicular malondialdehyde level. West Afr J Med. 2007;26(4):312-5.

9. Ekici S, Doğan Ekici AI, Öztürk G, Benli Aksungar F, Sinanoğlu O, Turan G, et al. Comparison of melatonin and ozone in the prevention of reperfusion injury following unilateral testicular torsion in rats. Urology. 2012;80(4):899-906.

10. Erdemir F, Parlaktaş BS, Özyurt H, Boztepe Ö, Atiş Ö, Şahin Ş. Antioxidant effect of melatonin in systemic circulation of rats after unilateral testicular torsion. Turkish Journal of Medical Sciences. 2008;38:1-6.

11. Eşrefoğlu M, Gül M, Parlakpinar H, Acet A. Effects of melatonin and caffeic acid phenethyl ester on testicular injury induced by myocardial ischemia/reperfusion in rats. Fundam Clin Pharmacol. 2005;19(3):365-72.

12. Gul SS, Gurgul S, Uysal M, Erdemir F. The Protective Effects of Pulsed Magnetic Field and Melatonin on Testis Torsion and Detorsion Induced Rats Indicated by Scintigraphy, Positron Emission Tomography/Computed Tomography and Histopathological Methods. Urol J. 2018;15(6):387-96.

13. Guo Y, Sun J, Li T, Zhang Q, Bu S, Wang Q, et al. Melatonin ameliorates restraint stress-induced oxidative stress and apoptosis in testicular cells via NF-κB/iNOS and Nrf2/ HO-1 signaling pathway. Sci Rep. 2017;7(1):9599.

14. Gurbilek M, Vatansev H, Gültekin F, Dilsiz A, Vatansev C, Aköz M. Prevention of testicular damage by free radical scavengers after acute experimental torsion. Biomedical Research. 2000;11:315-9.

15. Soma H, Mrinmoy S, Sananda D, Prasanta G, Sujay K. B, Debasish B, et al. Melatonin Ameliorates Heat Stress Induced Dysregulation of Testicular Function In Wistar Rat By Restoring Tissue Health, Hormone And Antioxidant Status And Modulating Heat Shock Protein Expression. International Journal of pharma and Bio Sciences. 2020.

16. Jeong SJ, Choi WS, Chung JS, Baek M, Hong SK, Choi H. Preventive effects of cyclosporine a combined with prednisolone and melatonin on contralateral testicular damage after ipsilateral torsion-detorsion in pubertal and adult rats. J Urol. 2010;184(2):790-6.

17. Kanter M. Protective effects of melatonin on testicular torsion/detorsion-induced ischemia-reperfusion injury in rats. Exp Mol Pathol. 2010;89(3):314-20.

18. Koksal M, Oğuz E, Baba F, Eren MA, Ciftci H, Demir ME, et al. Effects of melatonin on testis histology, oxidative stress and spermatogenesis after experimental testis ischemia-reperfusion in rats. Eur Rev Med Pharmacol Sci. 2012;16(5):582-8.

19. Kurcer Z, Oguz E, Ozbilge H, Baba F, Aksoy N, Celik N. Effect of melatonin on testicular ischemia/reperfusion injury in rats: is this effect related to the proinflammatory cytokines? Fertil Steril. 2008;89(5 Suppl):1468-73.

20. Kurcer Z, Hekimoglu A, Aral F, Baba F, Sahna E. Effect of melatonin on epididymal sperm quality after testicular ischemia/reperfusion in rats. Fertil Steril. 2010;93(5):1545-9.

21. Mahmudi SAA, Ghasemi Hamidabadi H, Moayeri A, Nazm Bojnordi M, Zahiri M, Madani Z, et al. Melatonin ameliorates testes against forced treadmill exercise training on spermatogenesis in rats. Folia Med (Plovdiv). 2022;64(1):75-83.

22. Minaii B, Moayeri A, Shokri S, Habibi Roudkenar M, Golmohammadi T, Malek F, et al. Melatonin improve the sperm quality in forced swimming test induced oxidative stress in nandrolone treated Wistar rats. Acta Med Iran. 2014;52(7):496-504.

23. Mirhoseini M, Talebpour Amiri F, Karimpour Malekshah AA, Rezanejad Gatabi Z, Ghaffari E. Protective effects of melatonin on testis histology following acute torsion-detorsion in rats. Int J Reprod Biomed. 2017;15(3):141-6.

24. Mirhoseini M, Rezanejad Gatabi Z, Saeedi M, Morteza-Semnani K, Talebpour Amiri F, Kelidari HR, et al. Protective effects of melatonin solid lipid nanoparticles on testis histology after testicular trauma in rats. Res Pharm Sci. 2019;14(3):201-8.

25. Moayeri A, Mokhtari T, Hedayatpour A, Abbaszadeh HA, Mohammadpour S, Ramezanikhah H, et al. Impact of melatonin supplementation in the rat spermatogenesis subjected to forced swimming exercise. Andrologia. 2018;50(3).

26. Olayaki LA, Alagbonsi IA, Abdulkadir HO, Idowu FO. Low dose of melatonin ameliorates cryptorchidism-induced spermatotoxicity in rats. Journal of the Anatomical Society of India. 2017;66(1):67-71.

27. Onur R, Semerciöz A, Orhan I, Yekeler H. The effects of melatonin and the antioxidant defence system on apoptosis regulator proteins (Bax and Bcl-2) in experimentally induced varicocele. Urol Res. 2004;32(3):204-8.

28. Ozturk A, Baltaci AK, Mogulkoc R, Ozturk B. The effect of prophylactic melatonin administration on reperfusion damage in experimental testis ischemia-reperfusion. Neuro Endocrinol Lett. 2003;24(3-4):170-2.

29. Parlaktas BS, Atilgan D, Ozyurt H, Gencten Y, Akbas A, Erdemir F, et al. The biochemical effects of ischemia-reperfusion injury in the ipsilateral and contralateral testes of rats and the protective role of melatonin. Asian J Androl. 2014;16(2):314-8.

30. Qin DZ, Cai H, He C, Yang DH, Sun J, He WL, et al. Melatonin relieves heat-induced spermatocyte apoptosis in mouse testes by inhibition of ATF6 and PERK signaling pathways. Zool Res. 2021;42(4):514-24.

31. Saalu LC, V.A.Togun, Oyewopo AO, Raji Y. Artificial Cryptorchidism and the Moderating Effect of Melatonin (N-acetyl. 5 methoxy tryptamin) in Sprague-Dawley Rats. Journal of Applied Sciences. 2006;6:2889-94.

32. Sahna E, Türk G, Atessahin A, Yilmaz S, Olmez E. Remote organ injury induced by myocardial ischemia and reperfusion on reproductive organs, and protective effect of melatonin in male rats. Fertil Steril. 2007;88(1):188-92.

33. Sekmenli T, Gunduz M, Öztürk B, Karabağlı P, Ciftci I, Tekin G, et al. The effects of melatonin and colchicine on ischemia-reperfusion injury in experimental rat testicular torsion model. J Pediatr Surg. 2017;52(4):582-6.

34. Semercioz A, Onur R, Ogras S, Orhan I. Effects of melatonin on testicular tissue nitric oxide level and antioxidant enzyme activities in experimentally induced left varicocele. Neuro Endocrinol Lett. 2003;24(1-2):86-90.

35. Semercioz A, Baltaci AK, Mogulkoc R, Avunduk MC. Effect of Zinc and Melatonin on Oxidative Stress and Serum Inhibin-B Levels in a Rat Testicular Torsion-Detorsion Model. Biochem Genet. 2017;55(5-6):395-409.

36. Vargas A, Bustos-Obregón E, Hartley R. Effects of hypoxia on epididymal sperm parameters and protective role of ibuprofen and melatonin. Biol Res. 2011;44(2):161-7.

37. Yildirim A, Akkus M, Nergiz Y, Baran OP. The effect of melatonin on ductus epididymis. Unilateral testicular torsion in rats. Saudi Med J. 2007;28(2):288-9.

38. Yuan XC, Wang P, Li HW, Wu QB, Zhang XY, Li BW, et al. Effects of melatonin on spinal cord injury-induced oxidative damage in mice testis. Andrologia. 2017;49(7).

39. Yurtçu M, Abasiyanik A, Avunduk MC, Muhtaroğlu S. Effects of melatonin on spermatogenesis and testicular ischemia-reperfusion injury after unilateral testicular torsion-detorsion. J Pediatr Surg. 2008;43(10):1873-8.

40. Yurtçu M, Abasiyanik A, Biçer S, Avunduk MC. Efficacy of antioxidant treatment in the prevention of testicular atrophy in experimental testicular torsion. J Pediatr Surg. 2009;44(9):1754-8.

41. Zhang P, Zheng Y, Lv Y, Li F, Su L, Qin Y, et al. Melatonin protects the mouse testis against heat-induced damage. Molecular Human Reproduction. 2020;26(2):65-79.
